# Bidirectional remodeling of β1-integrin adhesions during chemotropic regulation of nerve growth

**DOI:** 10.1186/1741-7007-9-82

**Published:** 2011-11-30

**Authors:** Lucas P Carlstrom, Jacob H Hines, Steven J Henle, John R Henley

**Affiliations:** 1 Medical Scientist Training Program, Mayo Graduate School, Mayo Clinic: College of Medicine, Rochester, Minnesota 55905, USA; 2 Department of Neurologic Surgery, Mayo Clinic: College of Medicine, Rochester, Minnesota 55905, USA; 3 Department of Physiology and Biomedical Engineering, Mayo Clinic: College of Medicine, Rochester, Minnesota 55905, USA; 4 Department of Pediatrics, University of Colorado Anschutz Medical Campus, Aurora, Colorado 80045, USA

## Abstract

**Background:**

Chemotropic factors in the extracellular microenvironment guide nerve growth by acting on the growth cone located at the tip of extending axons. Growth cone extension requires the coordination of cytoskeleton-dependent membrane protrusion and dynamic adhesion to the extracellular matrix, yet how chemotropic factors regulate these events remains an outstanding question. We demonstrated previously that the inhibitory factor myelin-associated glycoprotein (MAG) triggers endocytic removal of the adhesion receptor β1-integrin from the growth cone surface membrane to negatively remodel substrate adhesions during chemorepulsion. Here, we tested how a neurotrophin might affect integrin adhesions.

**Results:**

We report that brain-derived neurotropic factor (BDNF) positively regulates the formation of substrate adhesions in axonal growth cones during stimulated outgrowth and prevents removal of β1-integrin adhesions by MAG. Treatment of *Xenopus *spinal neurons with BDNF rapidly triggered β1-integrin clustering and induced the dynamic formation of nascent vinculin-containing adhesion complexes in the growth cone periphery. Both the formation of nascent β1-integrin adhesions and the stimulation of axon extension by BDNF required cytoplasmic calcium ion signaling and integrin activation at the cell surface. Exposure to MAG decreased the number of β1-integrin adhesions in the growth cone during inhibition of axon extension. In contrast, the BDNF-induced adhesions were resistant to negative remodeling by MAG, correlating with the ability of BDNF pretreatment to counteract MAG-inhibition of axon extension. Pre-exposure to MAG prevented the BDNF-induced formation of β1-integrin adhesions and blocked the stimulation of axon extension by BDNF.

**Conclusions:**

Altogether, these findings demonstrate the neurotrophin-dependent formation of integrin-based adhesions in the growth cone and reveal how a positive regulator of substrate adhesions can block the negative remodeling and growth inhibitory effects of MAG. Such bidirectional remodeling may allow the growth cone to rapidly adjust adhesiveness to the extracellular matrix as a general mechanism for governing axon extension. Techniques for manipulating integrin internalization and activation state may be important for overcoming local inhibitory factors after traumatic injury or neurodegenerative disease to enhance regenerative nerve growth.

## Background

Neurons form targeted synaptic connections during embryonic development of the nervous system. Dysfunction of this wiring can lead to severe neurologic disorders, including schizophrenia [1], autism [2] and mental retardation [3]. To make precise connections, neuron cell bodies project axons that navigate the environment to reach appropriate targets [4,5]. The axonal growth cone guides this pathfinding by detecting multiple diffusible and substrate bound cues; transducing guidance signals; and dynamically coordinating local responses that include rapid membrane retrieval and insertion events, the activation of cytoskeleton-dependent membrane protrusion and substrate adhesion machinery [6-10]. Inhibition of axon outgrowth and disrupted rewiring of synaptic connections underlies the inability of the central nervous system to functionally regenerate after injury [11,12]. The potent inhibitory factor myelin-associated glycoprotein (MAG), which is released after nervous system injury [13-15], was found recently to negatively regulate growth cone adhesions [16]. Remarkably, the growth inhibitory factors semaphorin 3A [17,18], Nogo-A [19,20] and chondroitin sulfate proteoglycans [21,22] also disrupt growth cone adhesions by distinct mechanisms. In general, the negative remodeling of adhesions by growth inhibitory cues can occur by dismantling adhesion complexes, by deactivating and internalizing integrin adhesion receptors and by inhibiting adhesion-dependent signaling [23-26].

Integrins are type I transmembrane receptors that function as intermediaries between extracellular matrix components and the actin cytoskeleton [27,28]. β1-integrin is highly expressed in motor and sensory neurons during development, but levels steadily decline into adulthood [29,30], where some expression is maintained for synaptic plasticity and stability [31]. Accumulating evidence indicates that integrins play an essential role in axon outgrowth and may serve as an important target for regenerative strategies [32-35]. Integrin receptors function bidirectionally through 'inside-out' and 'outside-in' mechanisms. Rearrangement of the integrin headpiece to a higher affinity ligand-binding state, known as integrin activation, is referred to as 'inside-out' signaling. Stimulating clustering in sphingolipid microdomains within the plasma membrane also increases integrin receptor avidity [36,37]. Intracellular signaling cascades initiated through integrin-ligand interactions refer to an 'outside-in' mechanism. Both inside-out and outside-in functions are mediated through cytoplasmic adhesion components. Integrin-based adhesion complexes consist of membrane-cytoskeletal and signaling proteins, such as vinculin, which acts to couple integrin receptors to actin filaments [38,39], talin, which induces integrin activation [40-42] and focal adhesion kinase (FAK), which phosphorylates various targets and regulates dynamic adhesion turnover to stimulate growth cone migration [25,43,44].

Neurotrophins are target-derived secreted factors that bind cognate Trk receptors expressed by the growth cone to promote neuron survival, differentiation, axon growth and synaptic plasticity [45-48]. The neurotrophin brain-derived neurotrophic factor (BDNF) binds and activates the receptor tyrosine kinase TrkB to control cytoskeletal rearrangements, membrane protrusion and growth cone motility [49-51]. How BDNF regulates integrins and adhesion complexes in the growth cone has remained incompletely defined [52-56]. Moreover, BDNF showed early promise as a central neural regenerative agent but, to date, no effective therapy has been developed that reliably stimulates functional recovery after a major human central nervous system event, such as a spinal cord injury [57-59]. Thus, elucidating the actions of BDNF on nerve growth and adhesion regulation in the combined presence of an inhibitory cue is of great interest.

Here, we report that BDNF induces the formation of nascent adhesion complexes in the growth cone that are necessary for the stimulation of axon growth by BDNF, as revealed by the sphingolipid L-*t*-lactosylceramide (L-*t*-LacCer), an inhibitor of lipid microdomain formation and integrin signaling, and through the use of a β1-integrin specific function blocking antibody. Moreover, we tested the impact of BDNF and MAG in combination, discovering that BDNF-induced adhesions are protected from disruption by secondary MAG exposure, whereas non-clustered integrins remain susceptible to internalization. This finding provides mechanistic insight into a previous report that priming neurons with a neurotrophin before MAG exposure restored neurite lengths to near control levels [60]. In contrast, we found that prior exposure to MAG prevented BDNF-induced adhesion formation and abolished stimulated axon elongation. Taken together, this work lends support to the hypothesis that adhesion complexes are an important target for the development of effective neural regenerative therapies.

## Results

### BDNF induces β1-integrin clustering at the growth cone surface

**Figure 1:**
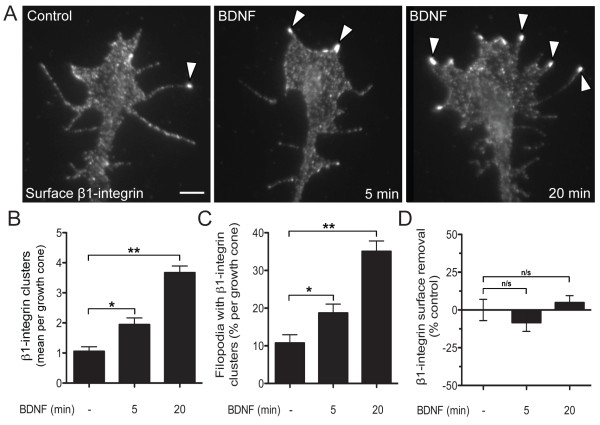
**BDNF stimulates β1-integrin clustering in the nerve growth cone**. **(A) ***Xenopus *spinal neuron growth cone immunolabeled for surface β1-integrin after treatment with vehicle alone (Control), or 5 min and 20 min BDNF (50 ng/mL) bath application. Arrowheads denote β1-integrin clusters. Scale bar, 5 μm. **(B, C) **Quantification of β1-integrin clustering after 5 min and 20 min BDNF treatment expressed as mean number of clusters per growth cone (B) and the percentage of growth cone filopodia that contain at least one β1-integrin cluster (C; see Methods). **(D) **Quantification of β1-integrin surface levels in the growth cone after 5 min and 20 min BDNF treatment (see Methods). Data are the mean ± standard error of the mean. (n > 200, n/s *P *> 0.05, *P < 0.01, **P < 0.001, ANOVA with Tukey's *post hoc *analysis.) BDNF: brain-derived neurotrophic factor.

Based on previous findings that growth inhibitory factors negatively remodel growth cone adhesion complexes, we hypothesized that a neurotrophin can positively regulate the distribution of integrin receptors at the surface plasma membrane. Immunostaining unpermeablized *Xenopus *spinal neurons for surface β1-integrin demonstrated a relatively homogeneous, diffuse localization at the growth cone with few focal clusters of enhanced fluorescence (Figure 1A). When neurons were treated with BDNF (50 ng/mL) for 5 min, surface immunostaining revealed more numerous focal clusters of β1-integrin in the growth cone periphery, which were often concentrated at filopodial tips (Figure 1A). Extending the duration of BDNF treatment to 20 min induced further β1-integrin clustering, as determined by an increased number of focal β1-integrin clusters. Quantitative analysis of thresholded images (see Methods) revealed a significant increase in the number of β1-integrin clusters in the growth cone after BDNF treatment for 5 min (two-fold) and 20 min (three-fold) compared to vehicle treated controls (Figure 1B). Treatment with BDNF for 90 min induced β1-integrin clustering comparable to the 20-min treatment (data not shown). We performed quality control analysis of the β1-integrin clustering by randomly selecting 50 growth cones for reanalysis and testing a range of threshold values including two-, two and a half-, three- and four-fold above the background fluorescence (see Methods). Levels of β1-integrin clusters increased significantly after BDNF treatment at both 5 min and 20 min time points compared to controls for each of the threshold values tested, demonstrating the general robustness of the ordering to the threshold value (Additional file 1). The image thresholding at three times above background fluorescence likely underestimates the absolute number of focal clusters in the growth cone, as demonstrated by the quality control analysis, but enabled a useful and reproducible comparison of relative changes in β1-integrin clustering between treatment groups. This quantitative approach also showed that the percentage of filopodia containing at least one β1-integrin cluster increased after BDNF treatment (Figure 1C). In contrast, the overall surface levels of β1-integrin in the growth cone remained unchanged after BDNF administration (Figure 1D). Thus BDNF induces integrin clustering rather than regulating global surface levels.

### BDNF induces formation of nascent adhesions

**Figure 2:**
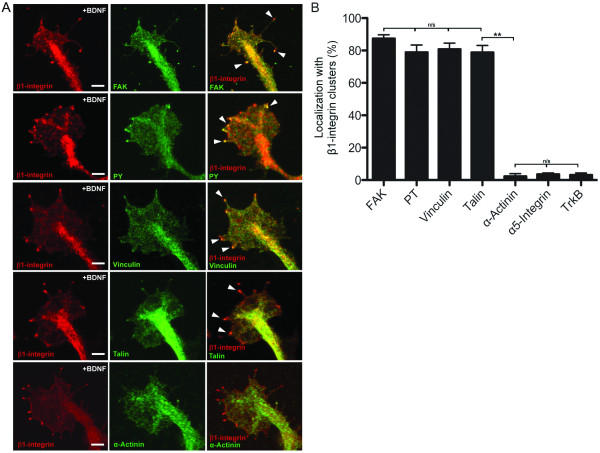
**BDNF triggers the formation of nascent growth cone adhesions**. **(A) **Representative confocal images of dual immunolabeled growth cones showing β1-integrin (red) and known cytoplasmic adhesion components (green: FAK; PY; vinculin; talin; α-actinin) after BDNF treatment (50 ng/mL; 20 min). Arrowheads in the merged images denote spots of co-localization. Scale bar, 5 μm. **(B) **Quantification of the percentage of total immunostaining for adhesion components, α5-integrin, and TrkB receptors that overlapped with β1-integrin clusters. Data are the mean ± standard error of the mean. (n > 40, n/s *P *> 0.05, **P < 0.001, ANOVA with Tukey's *post hoc *analysis.) BDNF: brain-derived neurotrophic factor; FAK: focal adhesion kinase; PY: phosphotyrosine.

To determine whether the β1-integrin clustering induced by BDNF localized within adhesion complexes, we performed double immunolabeling for both β1-integrin and known adhesion components. Treatment with BDNF induced β1-integrin clustering that localized together with puncta of increased FAK fluorescence in the growth cone periphery (Figure 2A). A similar distribution of FAK has been reported previously in these spinal neuron growth cones, consistent with its known scaffolding and signaling functions at sites of substrate adhesions [43,61]. The BDNF-induced integrin clusters also labeled positive for phosphotyrosine (PY), vinculin and talin, which are enriched in adhesion complexes. In contrast, α-actinin, a component of mature adhesions [62], was distinctly absent from the β1-integrin clusters. Quantifying the percentage of β1-integrin clusters that co-labeled for adhesion components revealed significant localization with FAK, phosphotyrosine, vinculin and talin but not α-actinin (Figure 2B). The BDNF receptor TrkB and α5-integrin were also absent from the β1-integrin puncta (Figure 2B). Overall, this distribution pattern is consistent with selective BDNF-induced clustering of β1-integrin within nascent adhesion complexes in the growth cone.

### Disrupting β1-integrin function blocks BDNF-induced clustering

**Figure 3:**
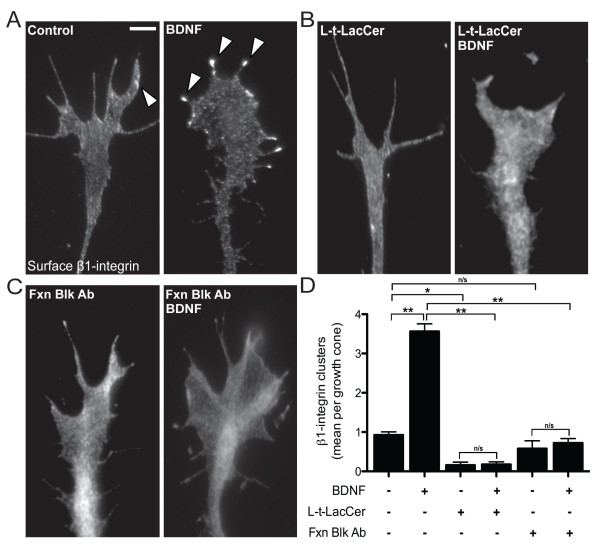
**BDNF-induced β1-integrin clustering requires intact lipid microenvironments and functional β1-integrin**. **(A) **Representative immunolabeled images showing β1-integrin after vehicle (BSA), BDNF (50 ng/mL; 20 min), **(B) **L-*t*-LacCer (20 μM) and L-*t*-LacCer plus BDNF, and **(C) **β1-integrin function blocking antibody (Fxn Blk Ab, 5 μg/mL) alone and plus BDNF treatments. Arrowheads designate clustered β1-integrins. Scale bar, 5 μm. **(D) **Quantification of β1-integrin clustering according to the treatment groups. Data are the mean ± standard error of the mean. (n > 150, **P < 0.001, ANOVA with Tukey's *post hoc *analysis.) BDNF: brain-derived neurotrophic factor; BSA: bovine serum albumin; L-*t*-LacCer: β-D-lactosyl-N-octanoyl-L-threo-sphingosine.

Integrin clustering is known to occur within glycosphingolipid (GSL)-enriched microdomains. We tested whether the synthetic GSL L-*t*-LacCer, which effectively disrupts GSL microdomains and inhibits integrin activation and signaling at the cell surface [63], might prevent β1-integrin clustering induced by BDNF. We first checked that cytoplasmic calcium ion (Ca^2+^) signaling, which is triggered in these spinal neuron growth cones by BDNF [64], is unperturbed by L-*t*-LacCer pretreatment. Live-cell Ca^2+ ^imaging revealed an elevation of cytoplasmic Ca^2+^, as detected by increased fluorescence (> 10%) of the Ca^2+^-sensitive indicator Fluo-8H, after BDNF treatment in four out of six growth cones imaged (Figure S2A in Additional file 2). In neurons pretreated with L-*t*-LacCer (20 μM; 30 min), BDNF induced a similar Ca^2+ ^elevation in six out of eight growth cones imaged (Figure S2B in Additional file 2). Both the maximal fluorescence intensity increase during the recording period and the maximal mean fluorescence intensity increase within a 3-min bin were similar in the BDNF alone and L-*t*-LacCer plus BDNF treatment groups (Figure S2C, D in Additional file 2). Thus, Ca^2+ ^signals downstream of BDNF treatment appear relatively normal in the growth cone after L-*t*-LacCer pretreatment. In contrast, pretreatment with L-*t*-LacCer reduced the surface β1-integrin clustering in the growth cone compared with vehicle treated controls and prevented clustering after a subsequent BDNF treatment (20 min; Figure 3A, B, D). The global surface levels of both β1-integrin and TrkB remained unchanged after treatment with L-*t*-LacCer compared to vehicle treated controls (Additional files 3 and 4).

We further addressed whether integrin function was necessary for β1-integrin clustering by utilizing the specific β1-integrin function-blocking antibody 2999 [65]. Pretreatment with antibody 2999 (5 μg/mL; 20 min) reduced the surface β1-integrin clustering in the growth cone compared to vehicle treated controls and prevented BDNF-stimulated β1-integrin clustering (Figure 3A, C, D). Total surface levels of both β1-integrin and TrkB were unchanged after treatment with antibody 2999 compared to vehicle treated controls (Additional files 3 and 4). Taken together, these findings support the notion that disrupting β1-integrin function blocks BDNF-dependent integrin clustering.

**Figure 4:**
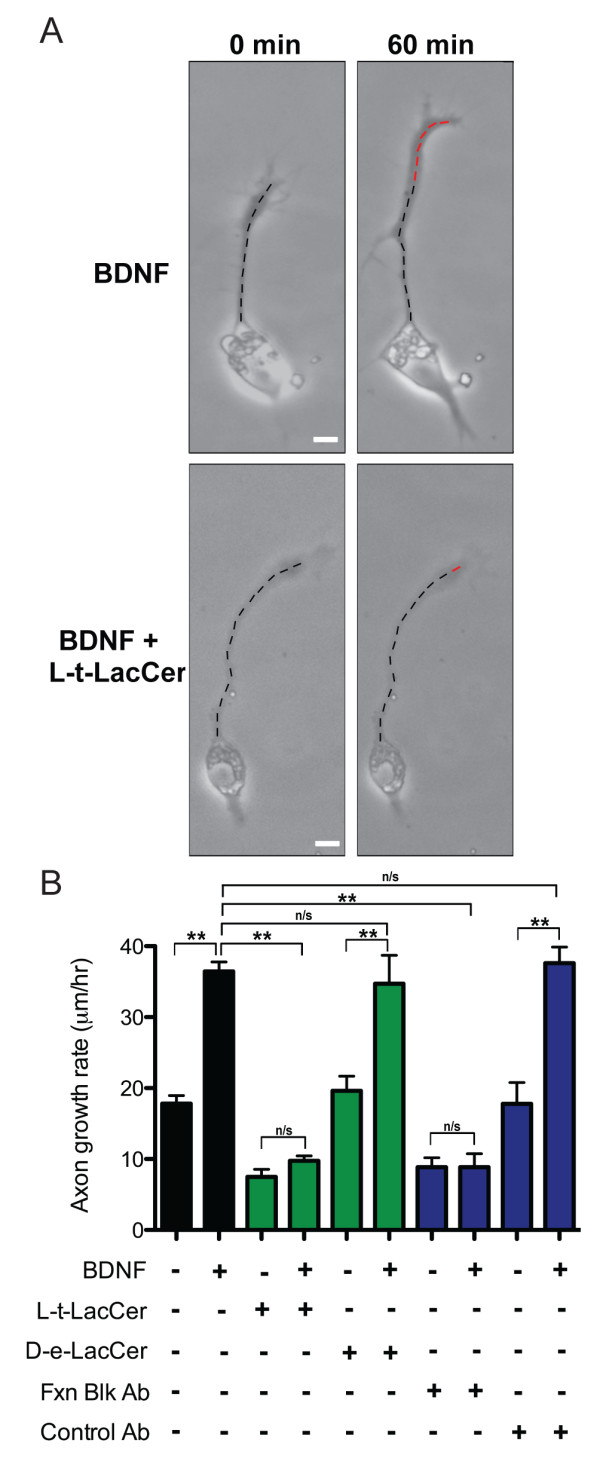
**Disrupting β1-integrin clustering impedes BDNF-dependent axon outgrowth**. **(A) **Axon growth assays with BDNF (50 ng/mL) or L-*t*-LacCer (20 μM) plus BDNF treatments demonstrating representative growth rates during a 60-min period. Scale bar, 10 μm. **(B) **Quantification of the axon growth rates of vehicle (BSA), BDNF, L-*t*-LacCer alone, L-*t*-LacCer plus BDNF, D-*e*-LacCer (20 μM), D-*e*-LacCer plus BDNF, β1-integrin function blocking antibody 2999 (Fxn Blk Ab; 5 μg/mL) alone and plus BDNF, control antibody (Control Ab, 5 μg/mL) and Control Ab plus BDNF treatments. Data are the mean ± standard error of the mean. (n > 150, **p < 0.001, ANOVA with Tukey's post hoc analysis). BDNF: brain-derived neurotrophic factor; BSA: bovine serum albumin; D-*e*-LacCer: D-lactosyl-β1-1'-N-octanoyl-D-*erythro*-sphingosine; L-*t*-LacCer: β-D-lactosyl-N-octanoyl-L-threo-sphingosine.

Is integrin clustering necessary for neurotrophin stimulation of neurite outgrowth? We addressed this by performing functional live-cell growth assays. Acute treatment with BDNF stimulated axon elongation during the 60-min assay as compared to vehicle treated controls (Figure 4A, B). Pretreatment with L-*t*-LacCer to disrupt integrin clustering before the growth assay reduced axon elongation compared with vehicle treated controls and impaired the BDNF-induced stimulation of outgrowth (Figure 4A, B). In contrast, pretreatment with the natural stereoisomer D-lactosyl-β1-1'-N-octanoyl-D-*erythro*-sphingosine (D-*e*-LacCer) permitted both normal basal outgrowth and the stimulation of outgrowth after a subsequent BDNF treatment (Figure 4B). Pretreatment with β1-integrin function-blocking antibody 2999 to prevent integrin clustering also impaired BDNF-stimulated axon elongation, whereas pretreatment with a control antibody permitted normal BDNF-dependent axon extension (Figure 4B). Altogether, these findings suggest that integrin clustering and activation are necessary for the stimulation of axon outgrowth by a neurotrophin.

We next tested whether treatment with either L-*t*-LacCer or the function-blocking antibody might impair growth cone membrane expansion, a stereotyped functional response to BDNF administration [66]. Molecular expression of a GFP-tagged chimera of paxillin, a cytoplasmic component of growth cone adhesions [67], demonstrated significant membrane expansion after BDNF treatment, as visualized by total internal reflection fluorescence (TIRF) microscopy to permit imaging exclusively at the ventral membrane contacting the substrate (Additional file 5). We also noted the rapid formation of numerous GFP-paxillin puncta consistent with nascent adhesions after BDNF treatment (Additional file 5). Measurements of growth cone diameter (see Methods) demonstrated that BDNF treatment caused significant widening of the growth cone compared with the pretreatment diameter (Figure S5A, B in Additional file 6). Significantly, neither the L-*t*-LacCer pretreatment nor the function-blocking antibody 2999 impaired the BDNF-induced growth cone expansion (Figure S5A, B in Additional file 6). Thus, both L-*t*-LacCer and the function-blocking antibody 2999 block BDNF-induced β1-integrin clustering and stimulation of axon outgrowth without disrupting BDNF signaling and downstream membrane expansion.

### BDNF-induced β1-integrin clustering is Ca^2+^-dependent

**Figure 5:**
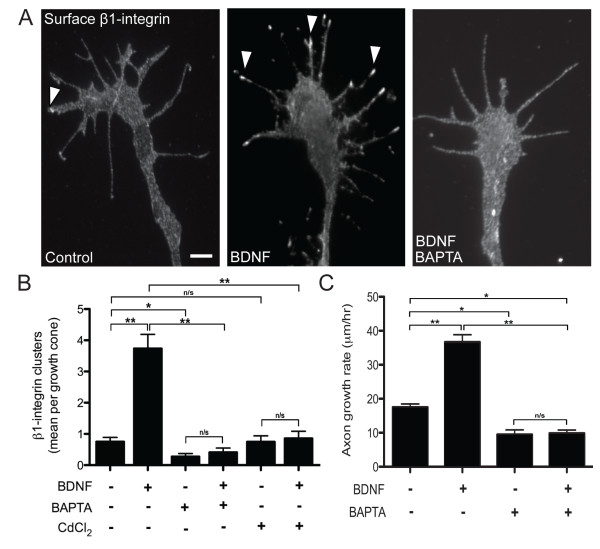
**BDNF-induced β1-integrin clustering and stimulated axon outgrowth is Ca^2+^-dependent**. **(A) **Representative immunolabeled growth cones showing the distribution of surface β1-integrin after treatments with vehicle (BSA), BDNF (50 ng/mL), or BAPTA-AM (1 μM; 30 nM [Ca^2+^]_e_) plus BDNF. Arrowheads denote β1-integrin clustering. Scale bar, 5 μm. **(B) **Quantification of β1-integrin clustering according to treatments with vehicle (BSA), BDNF (50 ng/mL), BAPTA-AM (1 μM; 30 nM [Ca^2+^]_e_) alone, BAPTA-AM (1 μM; 30 nM [Ca^2+^]_e_) plus BDNF, CdCl_2 _(50 μM; 20 min) alone, and CdCl_2 _plus BDNF. **(C) **Quantification of the mean axon growth rate after treatments with vehicle (BSA), BDNF (50 ng/mL), BAPTA-AM (1 μM; 30 nM [Ca^2+^]_e_), and BAPTA-AM (1 μM; 30 nM [Ca^2+^]_e_) plus BDNF. Data are the mean ± standard error of the mean. (n > 100, *P < 0.05, **P < 0.001, ANOVA with Tukey's *post hoc *analysis.) BDNF: brain-derived neurotrophic factor; BSA: bovine serum albumin; [Ca^2+^]_e_: extracellular Ca^2+ ^concentration.

Cytoplasmic Ca^2+ ^is an important second messenger for axon guidance signaling and is essential for positive growth cone chemotaxis towards a point source of BDNF in these spinal neurons [9,68]. To determine the role of Ca^2+ ^signaling during β1-integrin clustering by BDNF, we utilized the cell-permeant chelator BAPTA-AM, which effectively buffers cytoplasmic Ca^2+^. Surface immunolabeling revealed that pretreatment with BAPTA-AM completely blocked β1-integrin clustering induced by BDNF (Figure 5A, B). Pretreatment with 50 μM cadmium chloride (CdCl_2_) to nonselectively block voltage-dependent plasmalemmal Ca^2+ ^channels [69,70] also abolished the β1-integrin clustering induced by BDNF (Figure 5B). When spinal neurons were pretreated with BAPTA-AM during functional live-cell growth assays, the rate of constitutive axon elongation was reduced compared with normal Ca^2+ ^controls (Figure 5C). Importantly, BAPTA-AM treatment completely abolished the BDNF-stimulated growth rate compared to normal Ca^2+ ^controls. Taken together, these findings demonstrate that both β1-integrin clustering and stimulation of axon outgrowth by BDNF require intracellular Ca^2+ ^signaling.

### BDNF-induced β1-integrin adhesions resist downregulation by MAG

**Figure 6:**
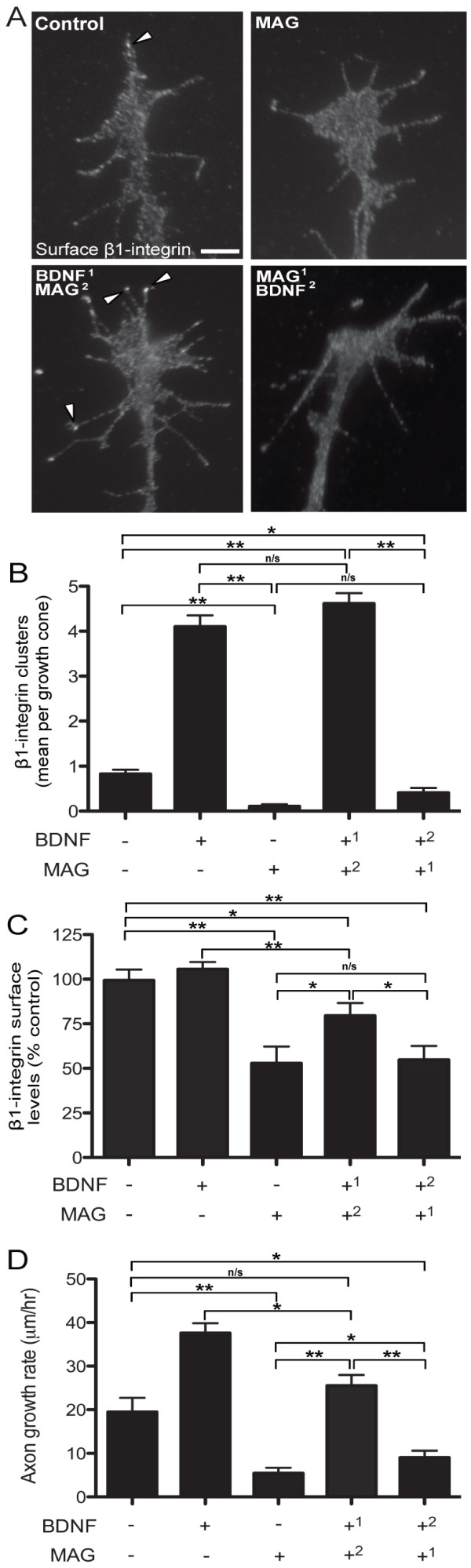
**BDNF priming counteracts inhibitory MAG-effects on β1-integrin clustering and growth inhibition**. **(A) **Representative immunolabeled images showing β1-integrin after control (BSA), MAG (1 μg/mL; 5 min), or combination treatments with BDNF (50 ng/mL; 20 min) and MAG. Detailed time course graphical representation of the combination treatments located in Additional file 8. Arrowheads designate clustered β1-integrins. Scale bar, 5 μm. **(B) **Quantification of β1-integrin clustering after vehicle (BSA), BDNF (50 ng/mL), and MAG (1 μg/mL) treatments alone or followed by secondary exposure to BDNF or MAG. **(C) **Quantification of β1-integrin surface levels after vehicle (BSA), BDNF (50 ng/mL), and MAG (1 μg/mL) treatments alone or followed by secondary exposure to BDNF or MAG. **(D) **Quantification of the mean axon growth rate according to the treatment groups. Data are the mean ± standard error of the mean. (n > 150, *P < 0.01, **P < 0.001, ANOVA with Tukey's *post hoc *analysis.) BDNF: brain-derived neurotrophic factor; BSA: bovine serum albumin; MAG: myelin-associated glycoprotein.

**Figure 7:**
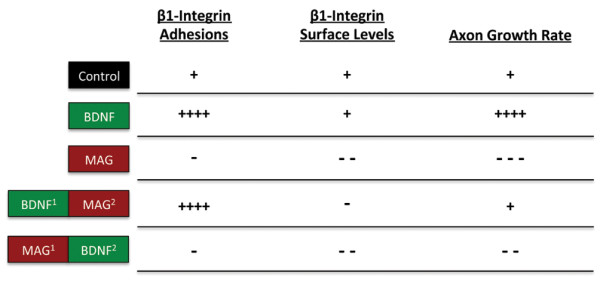
**Comparison of bidirectional β1-integrin remodeling during nerve growth**. Summary figure showing the clustering and global surface levels of β1-integrin in the growth cone correlated with the mean axon growth rate for the experimental treatments in Figure 6. Superscripts denote order of treatment. Symbols (+/-) denote positive and negative effects relative to controls.

We next tested whether the positive regulation of β1-integrin adhesions by BDNF might counteract negative remodeling by MAG. In growth cones exposed to MAG (1 μg/mL), surface immunostaining and quantitative analysis after image thresholding revealed a complete loss of clustered β1-integrin compared to vehicle treated controls (Figures 6A, B and 7). In contrast, pretreatment with BDNF (50 ng/mL) induced the formation of β1-integrin clusters that persisted even after a subsequent MAG exposure (Figures 6A, B and 7). We next asked whether BDNF might induce integrin clustering in the presence of MAG. In growth cones first exposed to MAG followed by a subsequent BDNF treatment, the β1-integrin clustering was abolished unlike the vehicle treated controls (Figures 6A, B and 7). Thus, BDNF priming induces β1-integrin clustering that can resist negative remodeling by MAG exposure, but secondary BDNF treatment cannot overcome the MAG-induced loss of clustering. Quality control analysis of the β1-integrin clustering using a range of threshold values including two-, two and a half-, three- and four-fold above the background fluorescence (see Methods), demonstrated statistical reliability for every data set except two experimental conditions that were analyzed with a threshold value four-fold above background fluorescence (Additional file 7).

Quantifying the mean surface β1-integrin immunostaining showed a reduction in global surface levels after MAG exposure alone compared with vehicle treated controls (Figures 6C and 7). Pretreatment with BDNF followed by MAG exposure partially protected the global surface levels of β1-integrin, which were elevated compared with MAG exposure alone, but were reduced compared with BDNF treatment alone (Figures 6C and 7). In growth cones first exposed to MAG followed by a subsequent BDNF treatment, the global surface levels of β1-integrin were reduced by levels comparable to those after MAG exposure alone (Figure 6C and 7). Altogether, these results suggest that the initial exposure to a positive or negative chemotropic cue may determine the level of β1-integrin clustering in the growth cone. Moreover, the mechanisms regulating integrin clustering can be distinct from those controlling the global level at the growth cone surface membrane.

Does the level of integrin clustering regulate axon extension? Pretreating rat cerebellar and dorsal root ganglion neurons with BDNF before plating onto MAG-expressing cells has been shown to block the growth inhibitory effects of chronic exposure to MAG in a 24-hour growth assay [60]. We tested for a correlation between the rate of axon extension and the direction of adhesion remodeling in the growth cone during acute exposure to MAG and BDNF. Treatment with BDNF stimulated the rate of axon extension during the 60-min functional assay, whereas single exposure to MAG-alone inhibited outgrowth compared to vehicle treated controls (Figures 6D and 7). In dual exposure assays, pretreatment with BDNF followed by MAG application resulted in a restoration of control levels of axon growth (Figures 6D and 7). On the other hand, initial MAG exposure followed by BDNF led to a relatively modest improvement in outgrowth over MAG treatment alone and was significantly reduced compared to control levels (Figures 6D and 7). Thus, the MAG-induced inhibition of axon extension correlates with negative β1-integrin clustering and reduced surface levels, and restoration of basal growth rate correlates with positive β1-integrin clustering and maintained surface levels. Stimulated outgrowth by BDNF may require both β1-integrin clustering and the complete protection of normal surface levels.

## Discussion

### Positive regulation of growth cone adhesions by BDNF

In a previous report, we demonstrated that a soluble form of MAG directs β1-integrin internalization to negatively modulate the functional distribution of integrin adhesions in the growth cone during repulsive guidance. Here, we have expanded on these findings by revealing that the neurotrophin BDNF positively regulates the formation of nascent integrin adhesions in the growth cone. The BDNF-induced clustering of β1-integrin localized to sites of substrate adhesions that co-labeled with *bona fide *adhesion components, including FAK, vinculin, phosphotyrosine and talin. The β1-integrin clustering was rapid, occurring during a 5-min BDNF treatment. Interestingly, the newly formed adhesions were negative for α-actinin, a component of more mature stable adhesion complexes, even after a 20-min BDNF treatment. These results are consistent with the notion that BDNF may stimulate both the formation and turnover of nascent adhesions, since stabile adhesions persisting for longer than 15 min would be expected to recruit α-actinin. Our finding, that β1-integrin clustering reached a new steady state during a 20-min BDNF treatment without any further increase after a 90-min treatment, further supports this idea. However, an alternative interpretation is that BDNF induces the formation of a distinct subclass of integrin adhesions in the growth cone that are α-actinin negative. While this manuscript was in review, a complementary finding was reported, that BDNF stimulates FAK-dependent formation and turnover of paxillin-containing substrate point contacts in the axonal growth cone during chemoattractive guidance [71]. This provided independent validation that BDNF induces nascent adhesions.

The BDNF-induced clustering of β1-integrin and stimulation of axon growth both required elevation of cytoplasmic Ca^2+^, a key second messenger downstream of TrkB activation, and functional lipid microdomains in the surface plasma membrane. How might local Ca^2+ ^signals facilitate integrin clustering and adhesion formation? It is well established that a rise in cytoplasmic Ca^2+ ^([Ca^2+^]_i_) leads to Ca^2+^/calmodulin-dependent protein kinases II (CamKII) activation [72,73]. One possibility is that CamKII activity positively regulates Tiam1 [74,75], Ras-GRF1/2 [76] and other guanine exchange factors to mediate Rho-family guanosine triphosphatase-dependent cytoskeletal dynamics and adhesion assembly [67,77]. An increase in [Ca^2+^]_i _has also been shown to trigger protein kinase C-mediated phosphorylation of Rho-guanosine nucleotide dissociation inhibitor, which then stimulates Rac localization to the plasma membrane [78]. Alternatively, [Ca^2+^]_i _elevation may initiate synaptotagmin I-mediated vesicular trafficking of adhesion receptors and components to focal sites at the plasma membrane, leading to increased adhesion formation and outgrowth [79]. Elucidating the signaling cascade initiated by BDNF that mediates integrin-based adhesion formation will be an important area of future investigation.

Many groups have now reported that the non-arbitrary, asymmetrically distributed lipids, including cholesterol and sphingolipids, influence integrin adhesion and signaling functions [36,80]. Specifically, the glycosphingolipid GM3 has been shown to directly regulate integrin-substrate interactions [81]. Additionally, talin and vinculin contain lipid binding sites that are thought to regulate adhesion formation and turnover [82]. Previous reports demonstrated that the synthetic GSL L-*t*-LacCer incorporates into the surface plasma membrane, disrupting lipid microdomains, integrin clustering and subsequent integrin activation [63]. Our finding, that L-*t*-LacCer disrupted β1-integrin clustering in the growth cone and attenuated the basal rate of axon outgrowth, is consistent with an essential role for the lipid microenvironment in normal integrin adhesion and signaling functions. This effect was more pronounced in the presence of BDNF, when L-*t*-LacCer pretreatment completely blocked β1-integrin clustering and prevented stimulation of axon extension. Our complementary results with the function-blocking antibody provide further support for the notion that BDNF-induced clustering of β1-integrin is essential for stimulated axon outgrowth. Altogether, our findings support a model whereby Ca^2+^-dependent signaling downstream of BDNF binding and TrkB activation induces the rapid clustering and formation of nascent β1-integrin adhesions in the growth cone to stimulate axon growth rate. Such dynamic remodeling may allow the growth cone to rapidly adjust adhesiveness to the extracellular matrix, providing a general mechanism for governing axon extension.

### Chemotropic effects determined by diametric regulation of growth cone adhesions

We showed previously that a diffusible microscopic gradient of MAG remodels growth cone adhesions asymmetrically during repulsive chemotactic guidance. Here, we have shown that the rapid global downregulation of functional β1-integrin adhesions at the growth cone surface membrane, induced by uniform exposure to MAG, correlates with the inhibition of axon extension in a similar time frame. The mechanism is Ca^2+^-dependent but distinct from the action of BDNF, since MAG triggers removal of β1-integrin from the surface membrane whereas total surface levels of β1-integrin remain unchanged by BDNF treatment. Thus, BDNF and MAG remodel growth cone adhesions in opposite directions during stimulation and inhibition of axon outgrowth, respectively. Importantly, the microenvironment of growth cone adhesions induced by BDNF protected against MAG-disruption and β1-integrin internalization. This BDNF priming did not block MAG-induced internalization of non-clustered β1-integrin, suggesting that the priming effect occurred by a mechanism other than cross-desensitization of receptors or second messengers. Internalization of the non-clustered integrin pool may partially account for the failure of BDNF priming to maximally stimulate nerve growth when MAG is present.

Treatment with BDNF after MAG application precludes adhesion formation and may provide a partial explanation for the relative lack of efficacy BDNF showed in human spinal cord regenerative efforts. We found that secondary BDNF treatment after MAG pre-exposure caused only a relatively modest improvement in the growth rate compared with MAG exposure alone, and was significantly attenuated compared with the control unperturbed growth rate. Neurotrophins can stimulate many cellular processes that are key for axon outgrowth. These include sprouting of new filopodia and membrane expansion at the growth cone, regulating actin dynamics and microtubule elongation, in addition to the formation of nascent substrate adhesions. Exposure to MAG may suppress many, but not necessarily all, of these actions. Taken together, our findings provide additional rationale for future studies aimed at manipulating the regulation of adhesion complexes in a translational regenerative model. We speculate that manipulations to disrupt the cycle of β1-integrin surface removal by MAG, such as inhibiting clathrin-mediated endocytosis or stimulating integrin activation and receptor redistribution, may potentially allow nerve growth stimulating agents like BDNF to positively regulate growth cone adhesions and encourage outgrowth through an inhibitory environment.

## Conclusions

This study demonstrates that the neurotrophin-induced formation of integrin-based adhesions in the growth cone, which is Ca^2+^-dependent, requires the clustering and activation of β1-integrin, and correlates with the stimulation of axon growth rate. This positive remodeling of integrin adhesions by BDNF opposes the negative remodeling induced by MAG during the inhibition of axon extension. Moreover, the BDNF-induced integrin clustering is resistant to negative remodeling by MAG, revealing a potential mechanism to explain how BDNF can block the growth inhibitory effects of MAG. Future therapeutic strategies targeted to manipulate dynamic integrin remodeling and activation state may be important for overcoming local inhibitory factors after traumatic injury or neurodegenerative disease to enhance neural regeneration.

## Methods

### Ethics statement

All animal experiments were carried out with strict adherence to National Institutes of Health (NIH) Guidelines for animal care and safety and were approved by the Mayo Clinic Institutional Animal Care and Use Committee.

### Primary cell culture of *Xenopus *spinal neurons

Wild-type *Xenopus laevis *(Xenopus One) were maintained in approved animal facilities according to institutional guidelines. Experiments were conducted on spinal neurons prepared from neural tube dissections of one-day old (stage 22) *X. laevis *embryos [83]. These cultures are used for experiments 10 h to 14 h after plating on fibronectin substrate at 20°C to 22°C. All cover glasses were coated with poly-D-lysine (5 mg/mL, Sigma, St. Louis, MO, USA) followed by fibronectin (20 μg/mL, Sigma). Culture medium consisted of 87.5% (v/v) Leibovitz medium (GIBCO, Grand Island, NY, USA) containing 0.4% (v/v) fetal bovine serum (HyClone, Logan, UT, USA), and 12.5% (v/v) saline solution (10 mM D-glucose, 5 sodium pyruvate, 1.26 mM calcium chloride (CaCl_2_) and 32 mM HEPES; pH 7.5). Experiments were performed in modified Ringers solution (120 mM sodium chloride (NaCl), 2.2 mM potassium chloride (KCl), 2 mM CaCl_2_, 1 mM magnesium chloride (MgCl_2_), 5 mM HEPES, 2 mM sodium pyruvate; pH 7.6). All animal research was performed with the approval of Mayo Clinic Institutional Animal Care and Use Committee.

### Reagents, immunolabeling and microscopy

Spinal neuron cultures were treated with MAG-Fc (1 μg/mL, R&D Systems #538-M, Minneapolis, MN, USA; conjugated to Fc-specific goat anti-human immunoglobulin G, Jackson IR Labs 109-485-098, West Grove, PA, USA), BDNF (50 ng/mL, Peprotech #450-02, Rocky Hill, NJ, USA), both or a control BSA vehicle solution for predetermined times, followed by standard chemical fixation. MAG treatments for 5 min were utilized to obtain maximal integrin surface removal [16]. Spinal neuron cultures were chemically fixed in a cytoskeleton-stabilizing buffer containing 2.5% paraformaldehyde and 0.01% glutaraldehyde for 20 min. All blocking and immunolabeling steps were performed in modified Ringers solution containing 5% goat serum. Alexa-dye-labeled secondary antibody conjugates (Invitrogen, Carlsbad, CA, USA) were used at 2 μg/mL. We immunolabeled unpermeabilized cells using a monoclonal antibody to the extracellular domain of β_1_-integrin (8c8-c, 0.8 μg/mL, University of Iowa Developmental Studies Hybridoma Bank) or with a β_1_-integrin function-blocking antibody (2999, 0.4 μg/mL, K. Yamada) along with a polyclonal anti-β-tubulin antibody (0.4 μg/mL, Abcam ab15568, Cambridge, England). Antibody staining for vinculin (2 μg/mL, Sigma V931), FAK (2 μg/mL, Santa Cruz Biotechnology sc-557, Santa Cruz, CA, USA), phosphotyrosine p-Tyr PY99 (1 μg/mL, Santa Cruz BioTechnology sc-7020), α5-integrin (1 μg/mL, D. DeSimone), TrkB (4 μg/mL, Novus NB100-92063, Littleton, CO, USA), α-actinin (1 μg/mL, Santa Cruz Biotechnology sc-59953) and talin (0.8 μg/mL, Abcam ab1188) was performed on permeabilized cells (0.1% Triton-X-100), followed by Alexa555 secondary antibody conjugates. Fluorescence microscopy was performed using a Zeiss (Jena, Germany) LSM 5LIVE confocal microscope equipped with a 63 × oil immersion objective (1.4 numerical aperture, 1.6 × optical zoom) with identical acquisition settings for control and experimental groups. The paxillin-GFP construct was provided by T.M. Gomez (University of Wisconsin, Madison). GFP-paxillin RNA was injected into four-celled *Xenopus *embryos. Spinal neurons were plated onto laminin (80 μg/mL) and incubated for 3 h to 4 h at 23°C to 25°C before use. High-magnification images were acquired using a Zeiss AxioCam MRm and 100 ×/1.45 NA objective on a Zeiss TIRF microscope.

### Image analysis and processing

To measure only receptors at the plasma membrane, permeabilized growth cones were excluded inadvertently from the analysis, as identified by tubulin immunofluorescence with polyclonal anti-β-tubulin. The original 14-bit images were analyzed using ImageJ (Bio-Formats ZVI plug-in, Madison, WI, USA). A region of interest encompassing the entire growth cone (defined as the distal 20 μm) was used to determine the mean fluorescence intensity of thresholded images (identical for experimental and control conditions). Data were background subtracted and normalized to the appropriate control images. Growth cones with a mean β1-integrin fluorescence level less than double the background fluorescence were excluded from analysis. For quantification of integrin clustering, we used a three-fold fluorescence inclusion criterion of β1-integrin cluster intensity over the mean background fluorescence in the growth cone central domain. Only clusters within the distal 20 μm of the axon and within 5 μm of the lateral borders of the growth cone (growth cone peripheral domain) were included. Quantification of β1-integrin clustering for quality control analysis was performed by randomly selecting growth cones (n = 50) for reevaluation with a range of fluorescence threshold values (two-, two and a half-, three-, and four-fold above the background fluorescence) [84]. To quantify co-labeling with β1-integrin puncta, a two-fold fluorescence increase relative to the mean fluorescence in the growth cone central domain was utilized for identifying cytoplasmic protein clusters. The growth cone diameter was determined using ImageJ by measuring from the lateral edges of lamellipodia at the widest point of the growth cone, excluding filopodia. The GFP-paxillin TIRF microscopy movie was made using ImageJ software by exporting time-lapse stacks to a QuickTime format (MOV, MPEG4 compression, 3 frames per second).

### Manipulation of β1-integrin clustering

We disrupted BDNF-induced integrin clustering using a 30-min pretreatment with the glycosphingolipid L-*t*-LacCer: β-D-lactosyl-N-octanoyl-L-threo-sphingosine (20 μM, Avanti Polar Lipids, Alabaster, AL, USA). The natural stereoisomer D-*e*-LacCer: D-lactosyl-β1-1'-N-octanoyl-D-erythro-sphingosine (20 μM, Avanti Polar Lipids) was used as a control lipid. Both L-*t*-LacCer and D-*e*-LacCer were complexed to defatted BSA and incubated with cells at a final concentration of 20 μM [63]. Integrin function was inhibited by a 20-min pretreatment with the function-blocking antibody 2999 (5 μg/mL). To buffer intracellular Ca^2+^, neuron cultures were incubated for 30 min in low-Ca^2+ ^(30 nM) solution consisting of 50% culture medium and 50% ethylene glycol tetraacetic acid (EGTA)-buffered saline (120 NaCl mM, 4.9 KCl mM, 1.55 mM MgCl_2_, 1.25 mM glucose, 5 mM sodium pyruvate, 4 mM HEPES, 0.65 mM EGTA, pH 7.6) and BAPTA-AM (1,2-bis-(o-aminophenoxy)-ethane-N, N, N', N'-tetraacetic acid, tetraacetoxymethyl ester, 1 μM; Calbiochem, Gibbstown, NJ, USA) or dimethyl sulfoxide vehicle for 30 min, followed by consecutive washes in low-Ca^2+ ^saline. To broadly disrupt Ca^2+ ^influx via voltage-dependent Ca^2+ ^channels in the plasmalemma, neurons were pretreated for 20 min with CdCl_2 _(50 μM, Sigma) as reported previously [70].

### Live-cell Ca^2+ ^imaging

*X. laevis *spinal neurons were loaded with the fluorescence Ca^2+ ^sensor Fluo-8H (2 μM, 30-min loading in culture medium containing 0.01% pluronic acid, AAT Bioquest, Sunnyvale, CA, USA). Growth cones were subjected to Ca^2+ ^imaging within 45 min of dye loading, using a Zeiss 200 M inverted microscope equipped with a 100X/1.45 NA objective and EM-CCD camera (Hamamatsu, Bridgewater, NJ, USA). Image acquisition was every 15 s and was started at least 2 min prior to application of BDNF (50 ng/mL). To measure fluorescence intensity, each series of images was thresholded to eliminate background noise, and the mean fluorescence intensity within a region of interest drawn around the growth cone was measured using ImageJ software (NIH). For each growth cone, the mean fluorescence intensity of each image after BDNF treatment (F) was then compared to the mean baseline fluorescence (pre-BDNF treatment; F_0_) to obtain the displayed value of normalized fluorescence (F/F_0_).

### Functional axon outgrowth assay

For measurements of neurite outgrowth in response to the various treatment groups, a series of time-lapse images were taken to record growth over a 60-min period (ProgRes CapturePro 2.7, Jenoptik Inc., Jupiter, FL, USA). Neurons were pretreated with L-*t*-LacCer or D-*e*-LacCer for 30 min in appropriate assays. A 20-min pretreatment was used for outgrowth assays with the function blocking antibody 2999 and a control antibody. All outgrowth assays were performed on a Zeiss 40 compact fluorescent lamp microscope equipped with a Ludl Electronic Products (Hawthorne, NY, USA) BioPoint 2 motorized stage, cooled charged-coupled device camera and a 20 × objective. Only axons > 50 μm in length were included in the analysis. Analysis was conducted using ImageJ.

### Statistical analyses

All statistical analyses were performed using GraphPad Prism software (v5, La Jolla, CA, USA). The figure legends state the statistical tests used. Data with a normal distribution (D'Agostino and Pearson omnibus normality test) were assessed using repeated-measures one-way analysis of variance with a Tukey *post hoc *analysis.

## Abbreviations

BDNF: brain-derived neurotrophic factor; BSA: bovine serum albumin; [Ca^2+^]_i_: intracellular Ca^2+ ^concentration; D-*e*-LacCer: D-lactosyl-β1-1'-N-octanoyl-D-*erythro*-sphingosine; EGTA: ethylene glycol tetraacetic acid; FAK: focal adhesion kinase; GFP: green fluorescent protein; GSL: glycosphingolipid; L-*t*-LacCer: β-D-lactosyl-N-octanoyl-L-threo-sphingosine; MAG: myelin-associated glycoprotein; TIRF microscopy: total internal reflection fluorescence microscopy.

## Authors' contributions

LPC, JHH and JRH conceived the project and designed experiments; LPC, JHH and SJH performed experiments and analyzed data; LPC and JRH wrote the manuscript; JRH supervised the project. All authors read and approved the final manuscript.

## Supplementary Material

Additional file 1 **Figure S1-Quality control analysis of β1-integrin clustering after time course BDNF treatments**. Growth cones from each experimental group in Figure 1B were randomly selected for quantification of β1-integrin clustering reanalysis (Research Randomizer V. 3.0 software), using a range of fluorescence threshold values (two-, two and a half-, three- and four-fold above the background fluorescence). Data are the mean ± s.e.m. (n = 50, *P < 0.05 as compared to control, ANOVA with Tukey's *post hoc *analysis.) Click here for file

Additional file 2 **Figure S2-BDNF-induced Ca^2+ ^signals after L-*t*-LacCer pretreatment**. **(A) **Representative images of the Fluo-8H Ca^2+ ^sensor in the growth cone before (left) and after (right) BDNF (50 μg/mL) treatment. Pseudocolor scale, blue = lower Ca^2+ ^and white = higher Ca^2+ ^concentrations. Scale bar, 5 μm. **(B) **As in (A) but with L-*t*-LacCer pretreatment. Scale bar, 5 μm. **(C and D) **Graphs showing the maximal fluorescence intensity after BDNF treatment normalized to the mean fluorescence in the pretreatment period (F/F_0_), with or without L-*t*-LacCer pretreatment (20 μM). The maximal F/F_0 _was measured at 1 time point (C) and during a binned 3-min period (D) for both conditions. Data are the mean ± s.e.m. (BDNF, n = 6; BDNF plus L-*t*-LacCer pretreatment, n = 8; *P < 0.05, t-test). Click here for file

Additional file 3 **Figure S3-Total β1-integrin surface levels are unaffected by treatments to disrupt β1-integrin clustering or Ca^2+ ^signaling**. Quantification of β1-integrin surface levels after vehicle (BSA), BDNF (50 μg/mL) alone, L-*t*-LacCer (20 μM) alone and plus BDNF, the β1-integrin function-blocking antibody (Fxn Blk Ab; 5 μg/mL) alone and plus BDNF, control antibody (5 μg/mL) alone and plus BDNF treatments. Data are the mean ± s.e.m. (n > 50, n/s *P *> 0.05, ANOVA with Tukey's *post hoc *analysis). Click here for file

Additional file 4 **Figure S4-Total TrkB surface levels are unaffected by treatments to disrupt β1-integrin clustering or Ca^2+ ^signaling**. Quantification of TrkB surface levels after vehicle (BSA), BDNF (50 μg/mL) alone, L-t-LacCer (20 μM) alone, L-t-LacCer plus BDNF, Fxn Blk Ab (5 μg/mL) alone, Fxn Blk Ab plus BDNF, control antibody (5 μg/mL) alone, and control antibody plus BDNF treatments. Data are the mean ± s.e.m. (n > 50, n/s *P *> 0.05, ANOVA with Tukey's *post hoc *analysis). Click here for file

Additional file 5 **Movie S1-BDNF-induced growth cone membrane expansion visualized through GFP-paxillin TIRF microscopy**. Representative time-lapse movie of a *Xenopus *spinal neuron growth cone expressing GFP-paxillin. Uniform bath application of BDNF (50 μg/mL; time 00:00) induced rapid membrane expansion. The TIRF images were collected every 1 min as indicated at the top left. Scale bar, 5 μm. Format: MOV (MPEG4 compression). Click here for file

Additional file 6 **Figure S5-BDNF-induced growth cone membrane expansion according to experimental treatments**. **(A) **Representative live-cell phase images of the growth cone during the pre- and post-treatment period with either vehicle (BSA), BDNF (50 μg/mL) alone, and L-*t*-LacCer (20 μM) plus BDNF. Dashed lines represent growth cone diameter measurement. Scale bar, 5 μm. **(B) **Quantification of the mean growth cone diameter during the pre- and post-treatment period with either vehicle (BSA), BDNF (50 μg/mL) alone, L-*t*-LacCer (20 μM) plus BDNF, the β1-integrin function-blocking antibody (Fxn Blk Ab; 5 μg/mL) alone and plus BDNF, BAPTA-AM (1 μM; 30 nM [Ca^2+^]_e_) alone and plus BDNF. Data are the mean ± s.e.m. (n > 50, *P < 0.05, ANOVA with Tukey's *post hoc *analysis). Click here for file

Additional file 7 **Figure S6-Quality control analysis of β1-integrin clustering after BDNF and MAG combination treatments**. Growth cones from each experimental group in Figure 6B were randomly selected for quantification of β1-integrin clustering reanalysis (Research Randomizer V. 3.0 software), using a range of fluorescence threshold values (two-, two and a half-, three- and four-fold above the background fluorescence). Data are the mean ± s.e.m. (n = 50, *P < 0.05, n/s *P *> 0.05 as compared to control, ANOVA with Tukey's *post hoc *analysis.) Click here for file

Additional file 8 **Figure S7-Graphical depiction of temporal treatment events for live-cell axon growth assays**. Summary figure showing the time-course sequence of combination treatments with L-*t*-LacCer, the β1-integrin function-blocking antibody (Fxn Blk Ab), BAPTA, MAG, and BDNF for the live cell axon growth rate assay used in Figures 3, 4, 5, 6. Click here for file
