# Supplementary figures and images for: Bidirectional remodeling of β1-integrin adhesions during chemotropic regulation of nerve growth
Source: BMC Biol. 2011 Nov 30;9:82. doi: 10.1186/1741-7007-9-82 (PMC3283487; doi:10.1186/1741-7007-9-82)

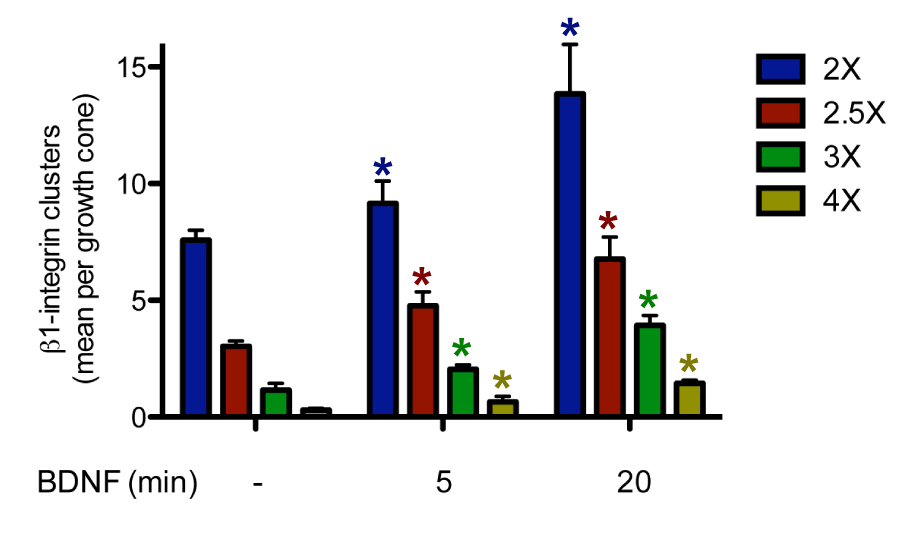

Supplement: Additional file 1 — Figure S1-Quality control analysis of β1-integrin clustering after time course BDNF treatments. Growth cones from each experimental group in Figure 1B were randomly selected for quantification of β1-integrin clustering reanalysis (Research Randomizer V. 3.0 software), using a range of fluorescence threshold values (two-, two and a half-, three- and four-fold above the background fluorescence). Data are the mean ± s.e.m. (n = 50, *P < 0.05 as compared to control, ANOVA with Tukey's post hoc analysis.) [file 1741-7007-9-82-S1.TIFF]

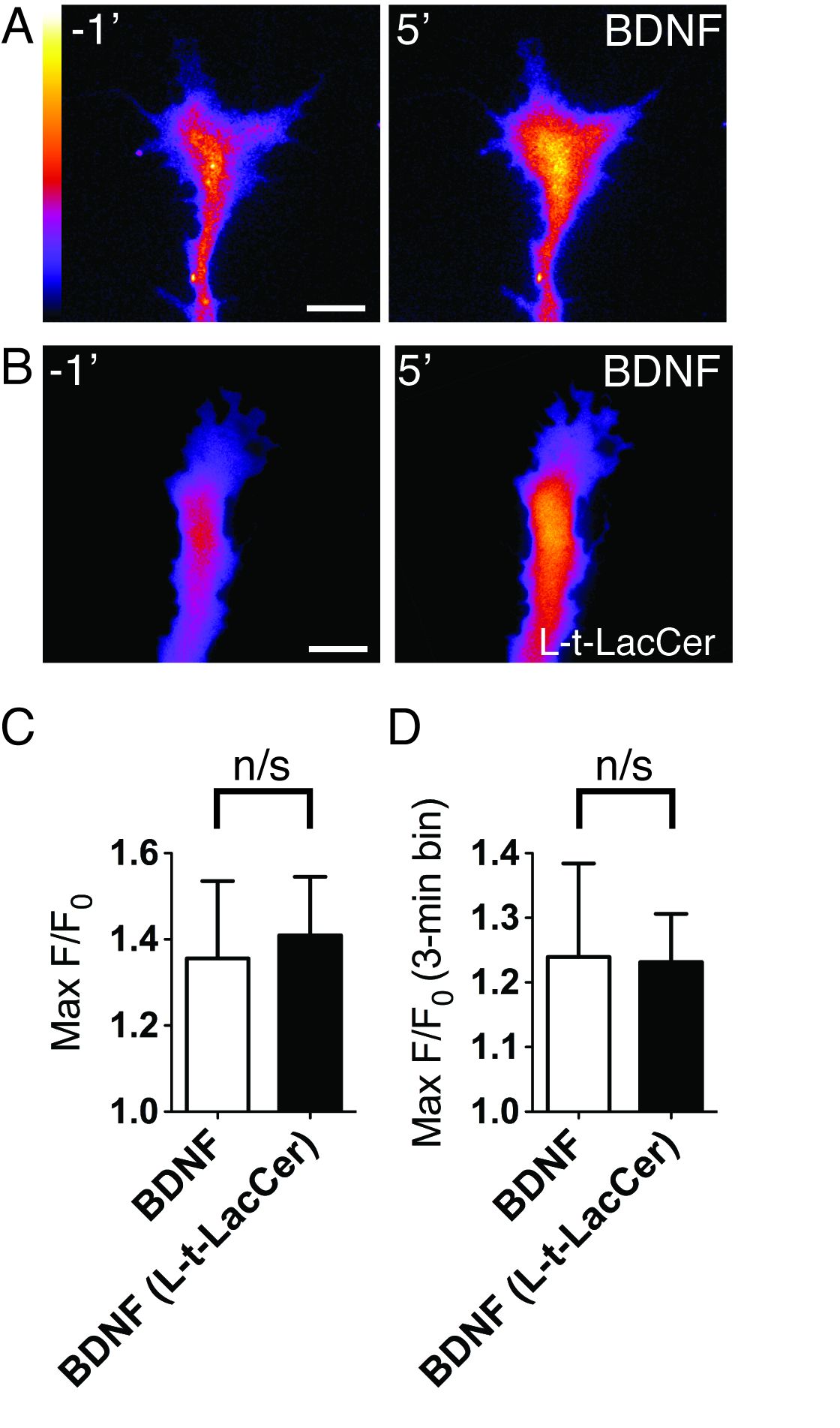

Supplement: Additional file 2 — Figure S2-BDNF-induced Ca2+ signals after L-t-LacCer pretreatment. (A) Representative images of the Fluo-8H Ca2+ sensor in the growth cone before (left) and after (right) BDNF (50 μg/mL) treatment. Pseudocolor scale, blue = lower Ca2+ and white = higher Ca2+ concentrations. Scale bar, 5 μm. (B) As in (A) but with L-t-LacCer pretreatment. Scale bar, 5 μm. (C and D) Graphs showing the maximal fluorescence intensity after BDNF treatment normalized to the mean fluorescence in the pretreatment period (F/F0), with or without L-t-LacCer pretreatment (20 μM). The maximal F/F0 was measured at 1 time point (C) and during a binned 3-min period (D) for both conditions. Data are the mean ± s.e.m. (BDNF, n = 6; BDNF plus L-t-LacCer pretreatment, n = 8; *P < 0.05, t-test). [file 1741-7007-9-82-S2.TIFF]

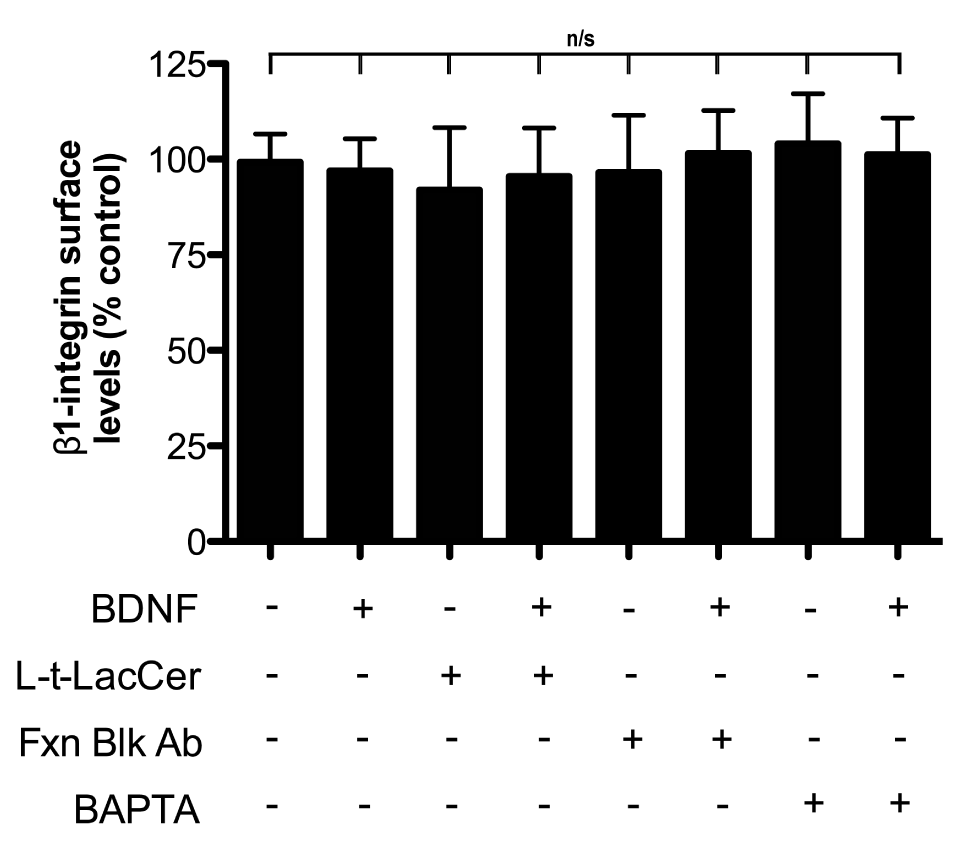

Supplement: Additional file 3 — Figure S3-Total β1-integrin surface levels are unaffected by treatments to disrupt β1-integrin clustering or Ca2+ signaling. Quantification of β1-integrin surface levels after vehicle (BSA), BDNF (50 μg/mL) alone, L-t-LacCer (20 μM) alone and plus BDNF, the β1-integrin function-blocking antibody (Fxn Blk Ab; 5 μg/mL) alone and plus BDNF, control antibody (5 μg/mL) alone and plus BDNF treatments. Data are the mean ± s.e.m. (n > 50, n/s P > 0.05, ANOVA with Tukey's post hoc analysis). [file 1741-7007-9-82-S3.TIFF]

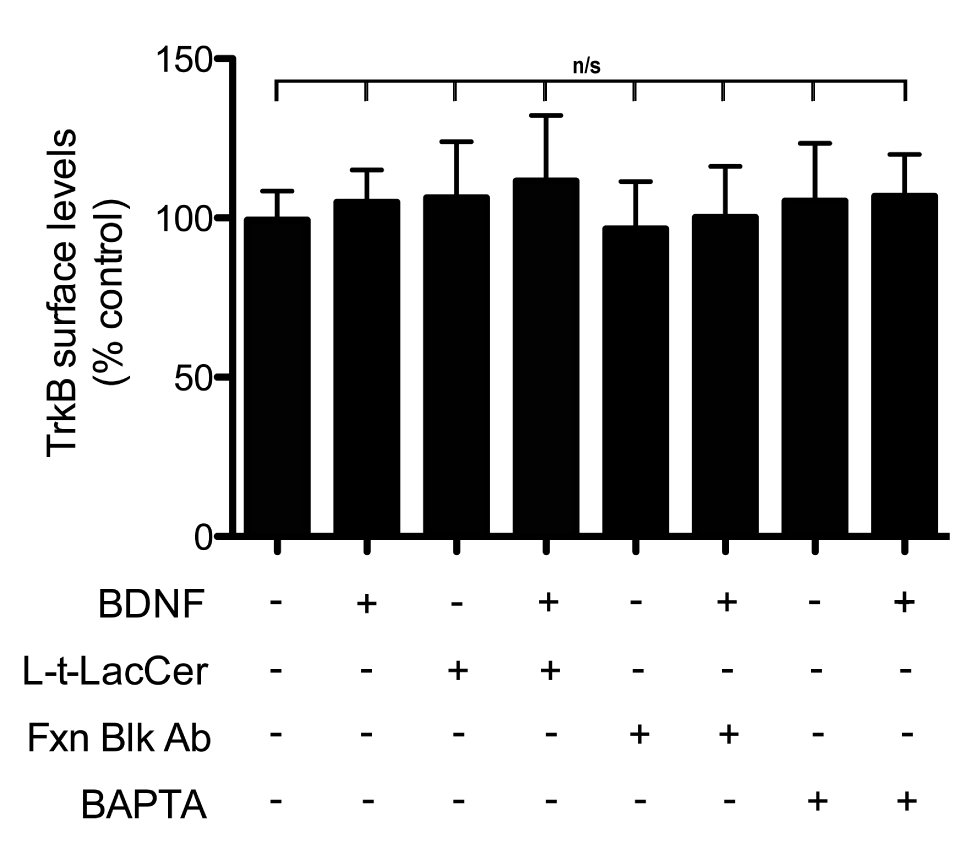

Supplement: Additional file 4 — Figure S4-Total TrkB surface levels are unaffected by treatments to disrupt β1-integrin clustering or Ca2+ signaling. Quantification of TrkB surface levels after vehicle (BSA), BDNF (50 μg/mL) alone, L-t-LacCer (20 μM) alone, L-t-LacCer plus BDNF, Fxn Blk Ab (5 μg/mL) alone, Fxn Blk Ab plus BDNF, control antibody (5 μg/mL) alone, and control antibody plus BDNF treatments. Data are the mean ± s.e.m. (n > 50, n/s P > 0.05, ANOVA with Tukey's post hoc analysis). [file 1741-7007-9-82-S4.TIFF]

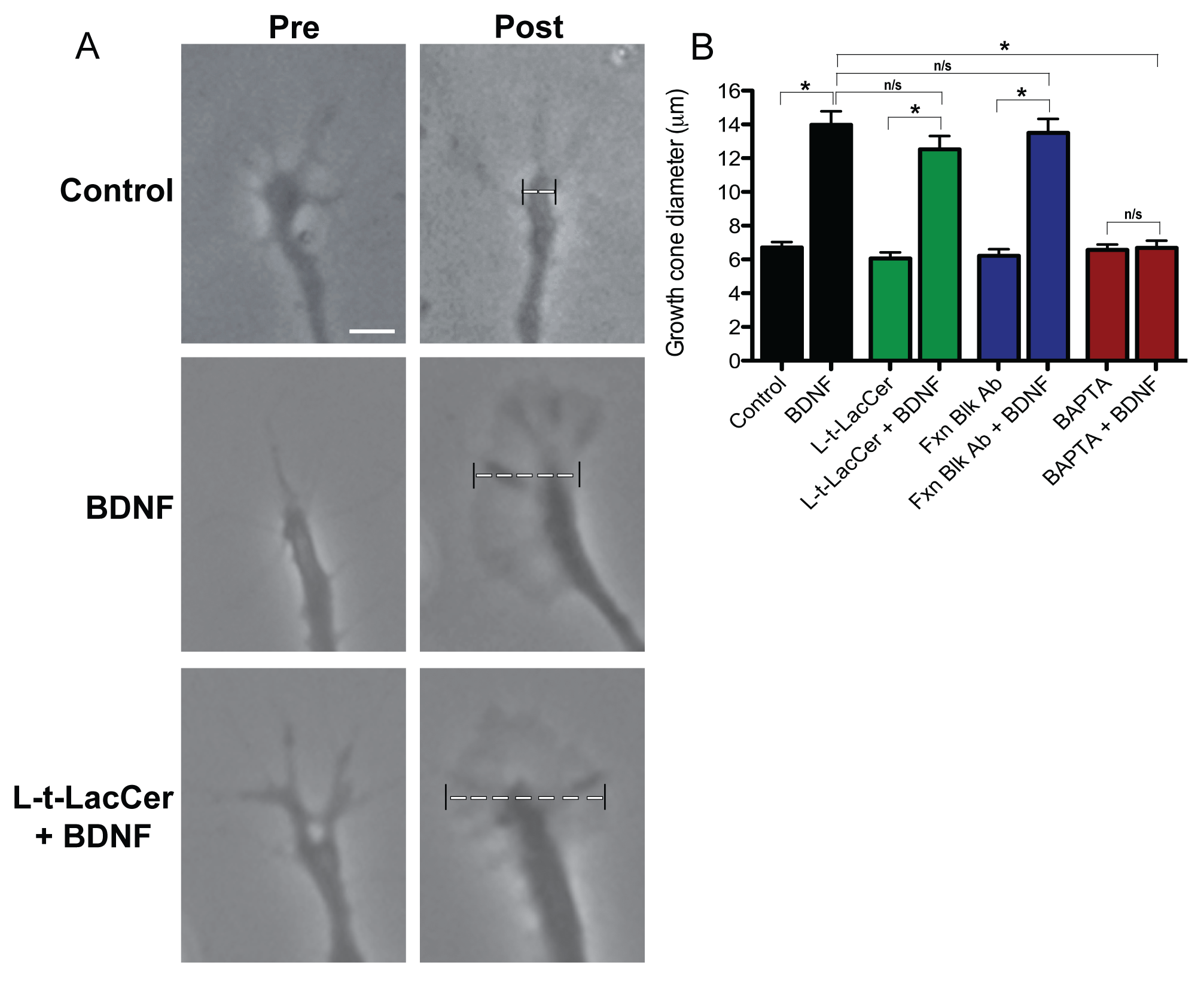

Supplement: Additional file 6 — Figure S5-BDNF-induced growth cone membrane expansion according to experimental treatments. (A) Representative live-cell phase images of the growth cone during the pre- and post-treatment period with either vehicle (BSA), BDNF (50 μg/mL) alone, and L-t-LacCer (20 μM) plus BDNF. Dashed lines represent growth cone diameter measurement. Scale bar, 5 μm. (B) Quantification of the mean growth cone diameter during the pre- and post-treatment period with either vehicle (BSA), BDNF (50 μg/mL) alone, L-t-LacCer (20 μM) plus BDNF, the β1-integrin function-blocking antibody (Fxn Blk Ab; 5 μg/mL) alone and plus BDNF, BAPTA-AM (1 μM; 30 nM [Ca2+]e) alone and plus BDNF. Data are the mean ± s.e.m. (n > 50, *P < 0.05, ANOVA with Tukey's post hoc analysis). [file 1741-7007-9-82-S6.TIFF]

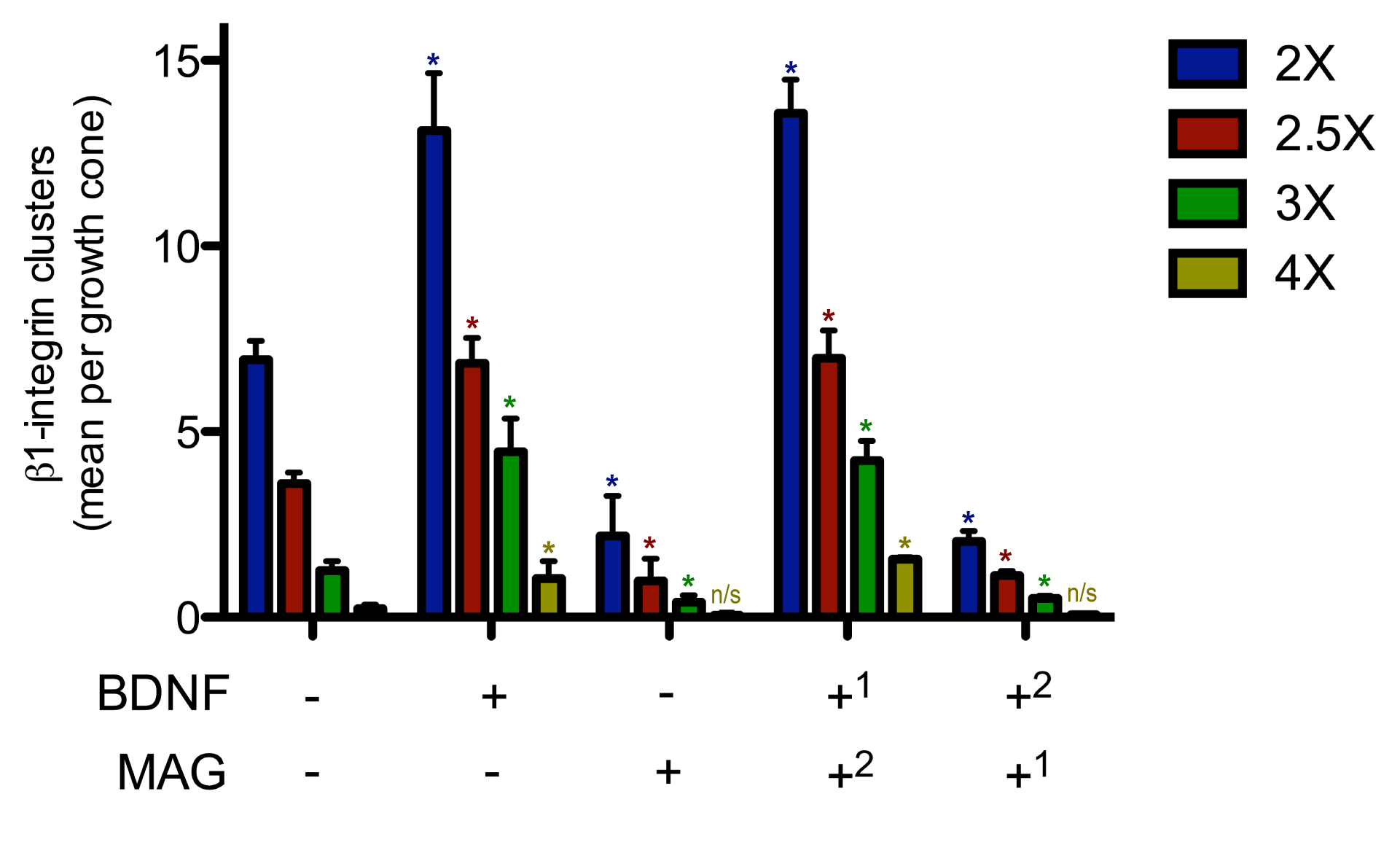

Supplement: Additional file 7 — Figure S6-Quality control analysis of β1-integrin clustering after BDNF and MAG combination treatments. Growth cones from each experimental group in Figure 6B were randomly selected for quantification of β1-integrin clustering reanalysis (Research Randomizer V. 3.0 software), using a range of fluorescence threshold values (two-, two and a half-, three- and four-fold above the background fluorescence). Data are the mean ± s.e.m. (n = 50, *P < 0.05, n/s P > 0.05 as compared to control, ANOVA with Tukey's post hoc analysis.) [file 1741-7007-9-82-S7.TIFF]

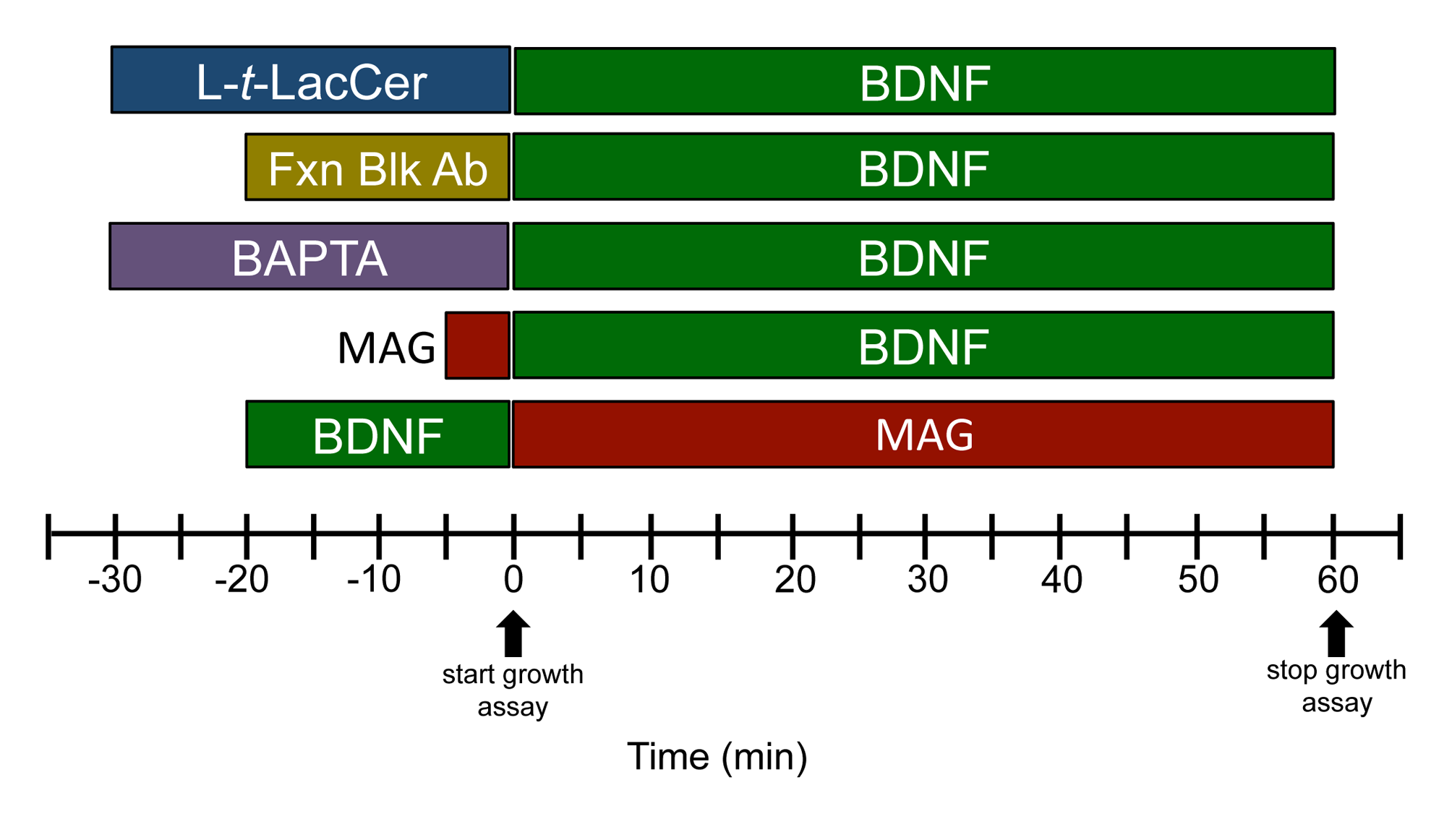

Supplement: Additional file 8 — Figure S7-Graphical depiction of temporal treatment events for live-cell axon growth assays. Summary figure showing the time-course sequence of combination treatments with L-t-LacCer, the β1-integrin function-blocking antibody (Fxn Blk Ab), BAPTA, MAG, and BDNF for the live cell axon growth rate assay used in Figures 3, 4, 5, 6. [file 1741-7007-9-82-S8.TIFF]
